# Silent Struggles: Uncovering Mental Health Burdens in Adolescents with Inflammatory Bowel Disease and Juvenile Idiopathic Arthritis—A Retrospective Chart Review

**DOI:** 10.3390/children12080995

**Published:** 2025-07-29

**Authors:** Kayla Beaudoin, Jaden Lo, Ethan Mewhinney, Kristen Bortolin, Tania Cellucci, Jenna Dowhaniuk, Liane Heale, Robert Issenman, Nikhil Pai, Mary Sherlock, Mary Zachos, Christina Grant, Karen Beattie, Katherine Prowse, Michelle Batthish

**Affiliations:** 1 Faculty of Health Sciences, McMaster University, Hamilton, ON L8S 4K1, Canada; 2 Division of Gastroenterology, Department of Pediatrics, McMaster University, Hamilton, ON L8S 4K1, Canada; 3 Division of Rheumatology, Department of Pediatrics, McMaster University, Hamilton, ON L8S 4K1, Canada; 4 Department of Gastroenterology, Hepatology & Nutrition, Children’s Hospital of Philadelphia, Philadelphia, PA 19104, USA; 5 Division of Adolescent Medicine, Department of Pediatrics, McMaster University, Hamilton, ON L8S 4K1, Canada

**Keywords:** mental health, youth, inflammatory bowel disease, juvenile idiopathic arthritis

## Abstract

**Highlights:**

**What are the main findings?**

- Most common mental health conditions in youth with IBD and JIA are generalized anxiety and major depression disorders;
- 12% of youth with IBD or JIA had more than one documented mental health diagnosis, and 14% take medication used to treat a mental health condition.

**What is the implication of the main finding?**

- Given these prevalences only represent those that are documented in the chart, this is likely an underrepresentation of the true prevalence of mental health conditions;
- Mental health screening should be considered in pediatric GI and rheumatology clinics to more accurately assess the burden of mental health conditions in youth.

**Abstract:**

Background/Objectives: Juvenile idiopathic arthritis (JIA) and inflammatory bowel disease (IBD) are chronic autoimmune conditions that impact the physical and psychological well-being of pediatric patients. While previous studies have shown a high prevalence of mental health challenges among youth with chronic conditions, the prevalence of mental health issues in Canadian pediatric patients with JIA and IBD remains unclear. We aimed to estimate the prevalence of documented mental health disorders and related medication use of youth with JIA or IBD at a tertiary care centre. Methods: We conducted a retrospective chart review of youths aged 12–17 diagnosed with JIA or IBD at McMaster Children’s Hospital (MCH) to understand the prevalence of generalized anxiety disorder (GAD), separation anxiety disorder, social anxiety disorder (SAD), obsessive–compulsive disorders (OCD), eating disorders, major depressive disorder (MDD), adolescent adjustment disorder, suicide attempt/suicide ideation, self-harm behaviour, substance use disorder, and attention deficit disorders (ADD). Results: We reviewed 429 patient charts, including 303 patients with IBD and 126 with JIA. Our findings identified 90 IBD patients and 20 JIA patients who had one or more documented mental health conditions. Proportionately, there was a higher prevalence of mental health conditions among IBD patients (30%) compared to JIA patients (16%). The most frequently observed conditions in both IBD and JIA patients were GAD (63%, 50%), ADD (33%, 35%), and MDD (29%, 15%). Conclusions: These findings highlight the critical need for early mental health screening and integrated care approaches that address both medical and psychosocial needs in adolescents with chronic illnesses. Future research should incorporate prospective study designs, include diverse geographic and demographic populations, and explore targeted interventions to improve mental and physical health outcomes in this vulnerable group.

## 1. Introduction

Juvenile idiopathic arthritis (JIA) and inflammatory bowel disease (IBD) are relapsing and remitting auto-immune diseases that pose significant physical and psychological challenges for pediatric patients. IBD encompasses two gastrointestinal (GI) conditions: ulcerative colitis, which affects colonic mucosa, and Crohn’s disease, which affects any region of the GI tract [1]. Canada has the highest prevalence rates of pediatric IBD worldwide, with approximately 38.25 per 100,000 children under the age of 16 affected [2]. JIA is chronic arthritis that develops in childhood and may be associated with other organ involvement, such as uveitis or skin rashes [3]. It is the most common type of arthritis in adolescents, with a prevalence rate of 3 in 1000 Canadian children [4].

Pediatric patients with IBD or JIA tend to experience more aggressive disease phenotypes, resulting in increased symptom severity, increased rates of surgical intervention and hospitalization, in addition to challenging treatment decisions and lower medication adherence [5,6,7]. When diagnosed during childhood, these conditions can disrupt growth, development, education, employment, vocational planning, and social behaviours, all of which are crucial for psychosocial development [7].

Not surprisingly, IBD and JIA can greatly impact an individual’s quality of life, as disease-related symptoms can impact physical, mental, and emotional well-being. Diagnostic procedures, hospital stays, and medication-related side effects also contribute to the stress experienced by patients and their families [8]. Symptoms such as abdominal pain, diarrhea, fatigue, and joint pain can be overwhelming [9], especially when they coincide with significant biological and psychosocial changes that occur during puberty and adolescence. There is considerable evidence linking chronic illnesses with mental health challenges in adolescents [10]. Studies indicate that youth with chronic conditions frequently report elevated levels of anxiety, depression, and stress, often due to the physical, social, and emotional demands of managing long-term health issues, compared to their peers without chronic illnesses [11,12]. Previous research highlights the interplay between mental and physical health, each capable of triggering or exacerbating issues in the other. The prevalence of mental health conditions correlates with the number of physical health issues [13,14]. Youth with chronic diseases experienced an even more significant decline in mental health compared to their peers without chronic conditions during the COVID-19 pandemic [15,16].

As a result, youth with IBD and JIA are at increased risk of developing mental health disorders. The unpredictable nature of chronic diseases, such as IBD or JIA, contributes to chronic stress, which can worsen both physical symptoms and psychological distress and, consequently, impact quality of life. Periods of heightened disease severity often bring physical discomfort, increased medical interventions, and interruptions to daily routines, all of which exacerbate anxiety, stress, and depressive symptoms. Furthermore, treatments for these chronic conditions may have side effects that impact mood and cognition; for example, systemic corticosteroid use has been associated with reduced cognitive functioning, insomnia, and an increased risk of mood and anxiety disorders [17].

Previous findings highlight the profound and multifaceted impact of chronic illness on adolescent mental health. In a Canadian cohort of adolescents with JIA, 40% of participants exhibited clinically significant symptoms of mental health disorders, with common diagnoses including major depressive disorder and panic disorder. Higher self-reported disease activity was also linked to heightened anxiety symptoms [18]. Similarly, adolescents with IBD face an increased risk of mental illness which has been shown to contribute to poorer disease outcomes, including worsened symptom control and diminished quality of life [19]. The COVID-19 pandemic further exacerbated these challenges, with youth affected by chronic physical conditions experiencing elevated psychological stress due to factors such as social isolation, uncertainty, and interruptions in health care access [20].

Despite these findings, the prevalence of mental health comorbidities has not been specifically examined post pandemic in youth with chronic conditions in Ontario. This study addressed this gap by determining the prevalence of documented mental health disorders in youth with IBD or JIA at McMaster Children’s Hospital (MCH) in Hamilton, Ontario, and the proportion of patients taking medications typically used to treat mental health conditions. Establishing this baseline is a critical first step toward understanding and addressing the mental health needs of pediatric patients with chronic inflammatory and arthritic diseases.

## 2. Materials and Methods

A list of all individuals aged 12–17 years old, diagnosed with IBD or JIA, who were receiving care in the pediatric gastroenterology or rheumatology clinics at MCH, and who had been seen in clinic at least once between June 2022 (start of hospital electronic medical record system (EMR)) and 29 December 2023, was generated. There were no exclusion criteria. Electronic medical records of each of these patients were reviewed by a research assistant.

When conducting electronic chart reviews, we extracted demographic information, including birth month and year, sex, and medical diagnosis (JIA, IBD, and IBD subtype where available). We also documented mental health conditions that were recorded in notes written by the treating gastroenterologist or rheumatologist or that were listed in the problem lists in the EMR. These conditions included the following diagnoses: generalized anxiety disorder (GAD), separation anxiety disorder, social anxiety disorder (SAD), obsessive–compulsive disorders (OCD), eating disorders—all, major depressive disorder (MDD), adolescent adjustment disorder, suicide attempt, suicide ideation, self-harm behaviour, substance use disorder, attention deficit disorders (ADD). All information was recorded in a de-identified data collection sheet.

For each participant, we also recorded all current medication names in the data collection sheet. Each medication name was then subsequently categorized into one of the following groups: non-steroidal anti-inflammatory drugs (NSAIDs), 5-aminosalicylic acid (5-ASA), immunomodulators, corticosteroids, biologics, antidepressants/anxiolytics, antipsychotics, and ADD medications.

To characterize the study population, we summarized continuous variables using means and standard deviations. Categorical variables were summarized using frequencies and proportions. The prevalence of each mental health condition was determined for the entire population and by disease subtype. We also determined the number/proportion of patients on medications related to mental health conditions. The Hamilton Integrated Research Ethics Board approved all study procedures.

## 3. Results

### 3.1. Patient Demographics

The number of eligible patients generated by our search criteria was 429 (Table 1). The mean age was similar in patients with IBD and JIA. There were more males and a higher prevalence of mental health conditions in patients with IBD compared to individuals with JIA (Table 1). There were 59 patients (47 IBD, 12 JIA) diagnosed with 1 mental health condition, 31 patients (25 IBD, 6 JIA) with 2, 13 patients (11 IBD, 2 JIA) with 3, and 8 patients (7 IBD, 2 JIA) with >3 mental health conditions.

### 3.2. Prevalence of Individual Mental Health Conditions

The prevalence of documented mental health conditions is summarized for the entire population and by disease in Table 2. The most common mental health conditions among IBD and JIA patients were GAD, MDD, and ADD (Table 2). The prevalence of GAD, MDD, ADD, suicide ideations, eating disorders, other, SAD, self-harm, separation anxiety disorder, OCD, and suicide attempt appeared similar between disease groups. There were 55 females (35%) and 56 males (21%) who had one or more documented mental health conditions.

### 3.3. Medications Used to Treat Mental Health Conditions

Medications used to treat mental health conditions were categorized into drug classes for the entire study population and by disease (Table 3). There were 35 (70%) patients (28 IBD, 7 JIA) prescribed only one medication for mental health, 10 (20%) patients (8 IBD, 2 JIA) and 5 (10%) patients (4 IBD, 1 JIA) prescribed two or three medications to treat a mental health condition, respectively. Additionally, there were 10 patients (9 IBD, 1 JIA) who were on a mental health-related medication but did not have a documented mental health condition in their EMR.

## 4. Discussion

This retrospective study assessed the prevalence of documented mental health conditions among adolescent patients with two chronic inflammatory conditions, IBD or JIA, at a single tertiary care centre in Ontario, Canada. Our findings indicated a higher prevalence of mental health conditions in IBD patients (30%) compared to JIA patients (15.8%), with an overall prevalence of 26%. GAD, MDD, and ADD were the most common mental health conditions in both IBD and JIA patients. Most patients with a comorbid mental health condition were treated with biologic medications (83 participants; 73 IBD, 10 JIA) and immunomodulators (24 participants; 15 IBD, 9 JIA). Antidepressants/anxiolytics were the most frequently prescribed mental health medications (41 participants; 32 IBD, 9 JIA).

The 30% prevalence of documented mental health conditions in patients with IBD and 16% in patients with JIA align with similar findings in adolescents with other chronic conditions. Roberts et al. reported a 31% mental health prevalence in a retrospective study of American adolescents and young adults aged 13–21 years old with type 2 diabetes [21]. In a prospective observational study of Korean pediatric patients younger than 18 years old with chronic kidney disease, Kang et al. reported a 20.5% prevalence of mental health, with 33.1% experiencing mental health or psychosocial adjustment problems [22]. Licari et al. performed a cross-sectional study and found that 43% of Italian adolescents aged 12–15 years old with asthma reported experiencing anxiety symptoms, while 18% reported depressive symptoms, both of which exacerbated asthma severity [23]. Additionally, Kyllönen et al. conducted a case-controlled cohort study and identified a 22.9% incidence rate of mental health conditions in 8–13-year-old JIA patients from Finland, highlighting their heightened risk of psychiatric morbidity [24]. Cooney et al. documented a 15.2% prevalence in IBD patients aged 5–25 years old across the United Kingdom and a 31.1% prevalence in individuals with IBD under 25 years in a retrospective study [25]. The prevalence rates found in our study may, in part, reflect the psychological impact of the COVID-19 pandemic, as Hawke et al. demonstrated significant declines in self-reported mental health among adolescents with physical health concerns during the pandemic [20].

The most common mental health conditions documented in the charts of our study population were GAD, MDD, and ADD, which are consistent with the existing literature. Cooney et al. identified depression, attention deficit disorders, and sleep disturbances as the most prevalent conditions among IBD patients, with an increased risk of developing post-traumatic stress disorder, eating disorders, self-harm, and other mental health challenges [25]. Whereas anxiety disorders (e.g., GAD, OCD, and panic disorders), mood disorders, and childhood behavioural conditions were found to be the most common mental health issues in JIA patients [24].

In this study, we focused exclusively on documented diagnoses of mental health conditions. Patients whose charts contained statements about symptoms or episodes without a documented diagnosis were not included. Consequently, our findings likely represent underestimates of diagnoses since the documentation of mental health conditions depends on health providers inquiring about mental health, patients disclosing this information to their medical team, and diagnoses being documented in medical records. Our findings highlight the importance of early mental health screening and monitoring for adolescents with chronic conditions. Coordinated care that integrates medical and mental health expertise has been shown to significantly reduce disease-related symptoms and enhance patients’ quality of life [26,27]. Supportive interventions, such as peer support groups and cognitive behavioural therapy, and follow-ups with primary care could address the unique psychosocial needs of this vulnerable population.

This study has several limitations. Its retrospective design restricted our ability to determine the exact timing of mental health diagnoses for participants. It is possible that some participants had pre-existing mental health conditions that were not recorded in their medical charts before we reviewed them. Additionally, underreporting or inconsistencies in medical records could impact the accuracy of the data, as this study was dependent on patients self-reporting mental health diagnoses and on physicians documenting these diagnoses.

Future research should consider prospective designs to establish the timing of diagnoses and track mental health status over time. Studies should also account for potential comorbidities, such as sleep disturbances and medication side effects, and include geographically diverse populations with various chronic conditions.

## 5. Conclusions

Adolescents with IBD or JIA face a significant mental health burden with a high prevalence of mental health conditions, particularly GAD, MDD, and ADD. This indicates a need for routine standardized mental health screening and for integrating multidisciplinary medical and mental health care for adolescents with chronic conditions.

## Figures and Tables

**Table 1:** Patient demographics.

Patient Characteristics	IBD (n = 303)	JIA (n = 126)	Total (n = 429)
Male sex, n (%)	192 (63%)	47 (37%)	239 (56%)
Age, mean [SD] years	14.7 [1.6]	14.5 [1.7]	14.7 [1.6]
Patients with ≥1 documented mental health conditions, n (%)	91 (30%)	20 (15.8%)	111 (26%)

JIA = juvenile idiopathic arthritis, IBD = inflammatory bowel disease, and SD = standard deviation.

**Table 2:** Mental health prevalence reveals differences between the IBD, JIA, and total patient groups. Mental health prevalence regarding GAD, ADD, MDD, suicide ideation, other, eating disorders, SAD, self-harm, separation anxiety disorder, obsessive–compulsive disorder, and suicide attempt.

Mental Health Condition	IBD (n = 91)	JIA (n = 20)	Total (n = 11)
GAD	57 (63%)	10 (50%)	67 (60%)
ADD	30 (33%)	7 (35%)	37 (33%)
MDD	26 (29%)	3 (15%)	29 (26%)
Suicide Ideation	16 (18%)	2 (10%)	18 (16%)
Other	10 (11%)	6 (30%)	16 (14%)
Eating Disorders	14 (16%)	0	14 (13%)
SAD	7 (8%)	0	7 (6%)
Self-Harm	5 (6%)	2 (10%)	7 (6%)
Separation Anxiety Disorder	3 (3%)	0	3 (3%)
Obsessive–Compulsive Disorder	3 (3%)	0	3 (3%)
Suicide Attempt	2 (2%)	0	2 (2%)

IBD = inflammatory bowel disease, JIA = juvenile idiopathic arthritis, GAD = generalized anxiety disorder, MDD = major depressive disorder, and ADD = attention deficit disorder.

**Table 3:** Medications in patients with documented mental health conditions.

Drug Class	IBD Patients (n = 91) on Medication n (%)	JIA Patients (n = 20) on Medication n (%)	Total Patients (n = 111) on Medication n (%)
JIA/IBD Medication			
Biologics	73 (80%)	10 (50%)	83 (75%)
Immunomodulators	15 (16%)	9 (45%)	24 (22%)
5-ASA	20 (22%)	0 (0%)	20 (18%)
Corticosteroids	19 (21%)	0 (0%)	19 (17%)
NSAIDs	0 (0%)	8 (40%)	8 (7%)
Biologics	73 (80%)	10 (50%)	83 (75%)
**Mental Health Medication**			
Antidepressants/ Anxiolytics	32 (35%)	9 (45%)	41 (37%)
ADD/ADHD medications	16 (18%)	5 (25%)	21 (19%)
Antipsychotics	9 (10%)	1 (5%)	10 (9%)

IBD = inflammatory bowel disease, JIA = juvenile idiopathic arthritis, NSAIDs = nonsteroidal anti-inflammatory drugs, and ADD = attention deficit disorder.

## Data Availability

The raw data supporting the conclusions of this article will be made available by the authors on request.

## Abbreviations

The following abbreviations are used in this manuscript:

ADD	Attention deficit disorder
GAD	Generalized anxiety disorder
GI	Gastrointestinal
IBD	Inflammatory bowel disease
JIA	Juvenile idiopathic arthritis
MCH	McMaster Children’s Hospital
MDD	Major depressive disorder
NSAIDs	Non-steroidal anti-inflammatory drugs
OCD	Obsessive–compulsive disorder
SAD	Social anxiety disorder
SD	Standard deviation
5-ASA	5-aminosalicyclic acid
